# Toll-like receptor 2–mediated NF-κB inflammatory responses in dry eye associated with cGVHD

**Published:** 2011-10-05

**Authors:** Chang He, Peilong Lai, Jianyu Weng, Shaoze Lin, Kaili Wu, Xin Du, Xialin Liu

**Affiliations:** 1 State Key Laboratory of Ophthalmology, Zhongshan Ophthalmic Center, Sun Yat-sen University, Guangzhou, P.R.China; 2 Department of Haematology, Guangdong General Hospital, Guangdong Academy of Medical Sciences, Guangzhou, P.R.China

## Abstract

**Purpose:**

Dry eye syndrome is one of the most common pathological manifestations in patients with chronic graft-versus-host disease (cGVHD). When dry eye occurs in association with cGVHD, the local inflammation is secondary to the systemic onset of inflammation. Toll-like receptor 2 (TLR2) signaling is thought to be essential for the inflammatory response and for immune disorders. The aim of this study was to explore whether the TLR2-mediated nuclear factor-kappa B (NF-κB) signaling pathway contributes to the inflammatory process of dry eye associated with cGVHD.

**Methods:**

Twenty patients with dry eye related to cGVHD and 20 controls were enrolled in this study. The patients with dry eye associated with cGVHD were diagnosed based on the clinical presentation and related ocular surface examinations such as Schirmer’s test and fluorescein staining. In addition, dry eye symptom scoring (National Institutes of Health consensus criteria) and Ocular Surface Disease Index (OSDI) scoring were also evaluated in all patients. Peripheral blood was collected from the patients and the controls for subsequent experiments. mRNA levels of *TLR2* and its downstream molecules, *NF-κB* and tumor necrosis factor (*TNF*)-α, were measured by quantitative Real-Time PCR (qRT–PCR). The protein level of TNF-α was detected by Enzyme-linked Immuno Sorbent Assay (ELISA).

**Results:**

*TLR2* mRNA in the peripheral blood from patients with dry eye related to cGVHD increased significantly compared with the controls. *NF-κB*, the downstream target of TLR2, also showed a marked elevation. The mRNA and protein levels of *TNF-α* were significantly upregulated in these patients. Importantly, the *TLR2* level was strongly correlated with the OSDI and the Schirmer’s test (r=0.565, p=0.010<0.05; r=0.564, p=0.016<0.05) in dry eye related to cGVHD compared with the controls.

**Conclusions:**

The TLR2-mediated NF–κB signaling pathway is activated in dry eye associated with cGVHD and contributes to the inflammatory state, which may predict the onset and progression of dry eye associated with cGVHD.

## Introduction

Chronic graft-versus-host disease (cGVHD) is a serious and common long-term complication of allogeneic hematopoietic stem cell transplantation (HSCT). Dry eye is one of the most common pathological manifestations of cGVHD, occurring in up to 80% of patients [1]. Dry eye associated with cGVHD can cause pain, ulcers or scars on the cornea, and even serious visual impairment, adversely affecting the performance of daily activities and impacting on the quality of life [2-4]. Effective therapy is very limited in this complex dry eye subgroup.

Studies have shown that dry eye associated with cGVHD is a prolonged inflammatory state, leading to progressive organic destruction [5-7]. At an early stage of the pathological process, donor T cells trigger antigen-presenting cells (APCs), including monocytes, dendritic cells, and natural killer cells, which have the potential to present antigens to naive T cells and activate effector T cells to produce a cytokine storm, leading to these cells homing to and attacking target tissue. Studies have indicated that a subclinical and complex cell-mediated immune or inflammatory reaction takes place in the conjunctiva of patients with dry eye secondary to cGVHD [8] but that the local inflammation of the ocular surface is secondary to the systemic onset of inflammation characterized by the cytokine storm [1,5-7,9]. However, the crucial signaling pathway that activates and mediates the systemic inflammatory response in dry eye associated with cGVHD remains to be fully elucidated.

Toll-like receptors (TLRs), which are a family of pattern recognition receptors, can provoke innate immunity and establish adaptive immunity, which can play a key role in APCs initiating an effector T cell response [10,11]. Toll-like receptor 2 (TLR2) on APCs can activate nuclear factor-kappa B (NF-κB), an inducible transcription factor of inflammatory signaling, and induce the production of inflammatory cytokines and costimulatory molecules, both of which are critical in autoimmunity and inflammation [12]. Many studies have reported a potential immune role for TLR2 signaling in the pathology of autoimmune-inflammatory diseases, such as systemic lupus erythematosus [13], arthritis [14], and inflammatory bowel disease [15]. Obviously, the pathological mechanism of dry eye associated with cGVHD involves an autoimmune-inflammatory abnormality. Thus, we hypothesized that the inflammatory process in dry eye associated with cGVHD might be mediated in part by activation of the immune pathway through TLR2.

In this study, we explored the expression of *TLR2*, as well as cytokines related to the TLR2–NF-κB signaling pathway, in dry eye associated with cGVHD. Our results showed that *TLR2* signaling was significantly activated in these patients, suggesting that *TLR2* contributes to the inflammatory state of dry eye secondary to cGVHD.

## Methods

### Patients

Twenty patients with prior HSCT who developed cGVHD with dry eye manifestations were enrolled in this study. Twenty patients who underwent HSCT but did not suffer from dry eye associated with cGVHD served as controls. The entire patients received bone marrow–sourced perfectly human leukocyte antigen matched stem cells. Dry eye associated with cGVHD was diagnosed when patients with established cGVHD diagnoses presented with symptoms and signs of dry eye disease according to the International Dry Eye WorkShop report, 2007 [16]. Other etiologies of dry eye, including Sjogren’s syndrome, Stevens-Johnson syndrome, and contact lens–related dry eye, were clinically excluded. Informed consent was obtained from all of the patients in accordance with the Declaration of Helsinki, and the protocol was approved by the Ethics Committee of Guangdong General Hospital.

### Observation and assessment of dry eye associated with cGVHD

It is difficult to evaluate the grades of dry eye associated with cGVHD due to the lack of uniform assessment criteria. In this study, we used the National Institutes of Health consensus criteria for organ scoring cGVHD to assess ocular manifestations. Scores of 0 (none) to 3 (worst) were assigned. Zero indicated no dry eye symptoms, 1 was defined as dry eye symptoms not affecting activities of daily living (ADL; eye drops ≤3 per day), 2 represented dry eye symptoms partially affecting ADL (eye drops >3 per day or punctal plugs) without vision impairment, and 3 indicated dry eye symptoms significantly affecting ADL (special eyewear to relieve pain) or making it impossible to work [17]. Additionally, we used the Ocular Surface Disease Index (OSDI) questionnaire to quantitatively evaluate the subjective severity of dry eye disease. The OSDI is a valid and reliable instrument for measuring the severity of dry eye disease. Scores ranged between 0 and 100, with higher scores representing more severe dry eye symptoms [18]. The specialized Schirmer’s test was performed to measure reflex tear secretion. Sterilized strips of filter paper were placed in the lateral canthus away from the cornea and left in place. Readings were reported in millimeters of wetting for 5 min [19].

### Quantitative real-time PCR analysis of *TLR2*, *NF-κB*, and *TNF-α* mRNA

Quantitative real-time PCR analysis was performed to detect the mRNA levels of *TLR2*, *NF-κB*, and *TNF-α* in peripheral blood mononuclear cells (PBMCs). Peripheral blood was collected by ethylenediamine tetraacetic acid anticoagulation, and the PBMCs were separated using the Ficoll-Hypaque gradient centrifugation method [20]. Total RNA was extracted with a Trizol kit (Invitrogen, Carlsbad, CA) and reversed into the first-strand cDNA by using random hexamer primers and the reverse transcriptase Superscript II Kit (Invitrogen) according to the manufacturer’s instructions. PCR was performed in a total volume of 20 μl containing 2 μl of cDNA, 10 μl of 2× SYBR Premix Ex Taq, 0.8 μl of 50× ROX Reference Dye (TaKaRa Biotechnology Co., Ltd, Dalian, PR, China), and 10 μmol/l primer pairs. The primers are shown in Table 1. The following cycling conditions were used: initial denaturation at 95 °C for 30 s, followed by 40 cycles at 95 °C for 5 s and 60 °C for 34 s according to the manufacturer’s instructions. The 2^(-ΔΔCT)^ method was used to present the data of the gene of interest relative to an internal control gene [21,22].

**Table 1:** Sequences of primers for Real-time PCR used in this study.

**Primers**	**Sequence**	**Function**
*ACTB*	5′-GCCAACACAGTGCTGTCTG-3′	Forward primer
	5′-TACTCCTGCTTGCTGATCCA-3′	Reverse primer
*TLR2*	5′-TGCTGCCATTCTCATTCTTCTG-3′	Forward primer
	5′-GCCACTCCAGGTAGGTCTTG-3′	Reverse primer
*NF-кB*	5′-TCTCGCCTGCCTCCACAAG-3′	Forward primer
	5′-TGTCTCCACGCCGCTGTC- 3′	Reverse primer
*TNF-α*	5′-ATCTACTCCCAGGTCCTCTTC-3′	Forward primer
	5′-GATGCGGCTGATGGTGTG-3′	Reverse primer

### ELISA analysis of TNF-α

Venous blood samples (1–2 ml) were collected from the patients with dry eye related to cGVHD and the controls. Levels of plasmatic TNF-α were measured by ELISA (Bender MedSystem, Vienna, Austria) according to the standard protocol provided by the manufacturer. The minimum detection level for the cytokine was 23 pg/ml. When the calculated cytokine concentration was below the given sensitivity, it was considered undetectable.

### Statistical analysis

Statistical analysis was performed using the SPSS13.0 software package (SPSS Inc., Chicago, IL). Data are shown as mean±standard deviation for separate experiments. The independent sample *t*-test analysis was used for the mRNA levels of *TLR2, NF-κB, TNF-α* and other indicators in both study and control group. Spearman’s rank correlation was computed to assess associations between the variables. A p value <0.05 was considered statistically significant.

## Results

### Clinical and pathological evaluation of dry eye associated with cGVHD

**Figure 1:**
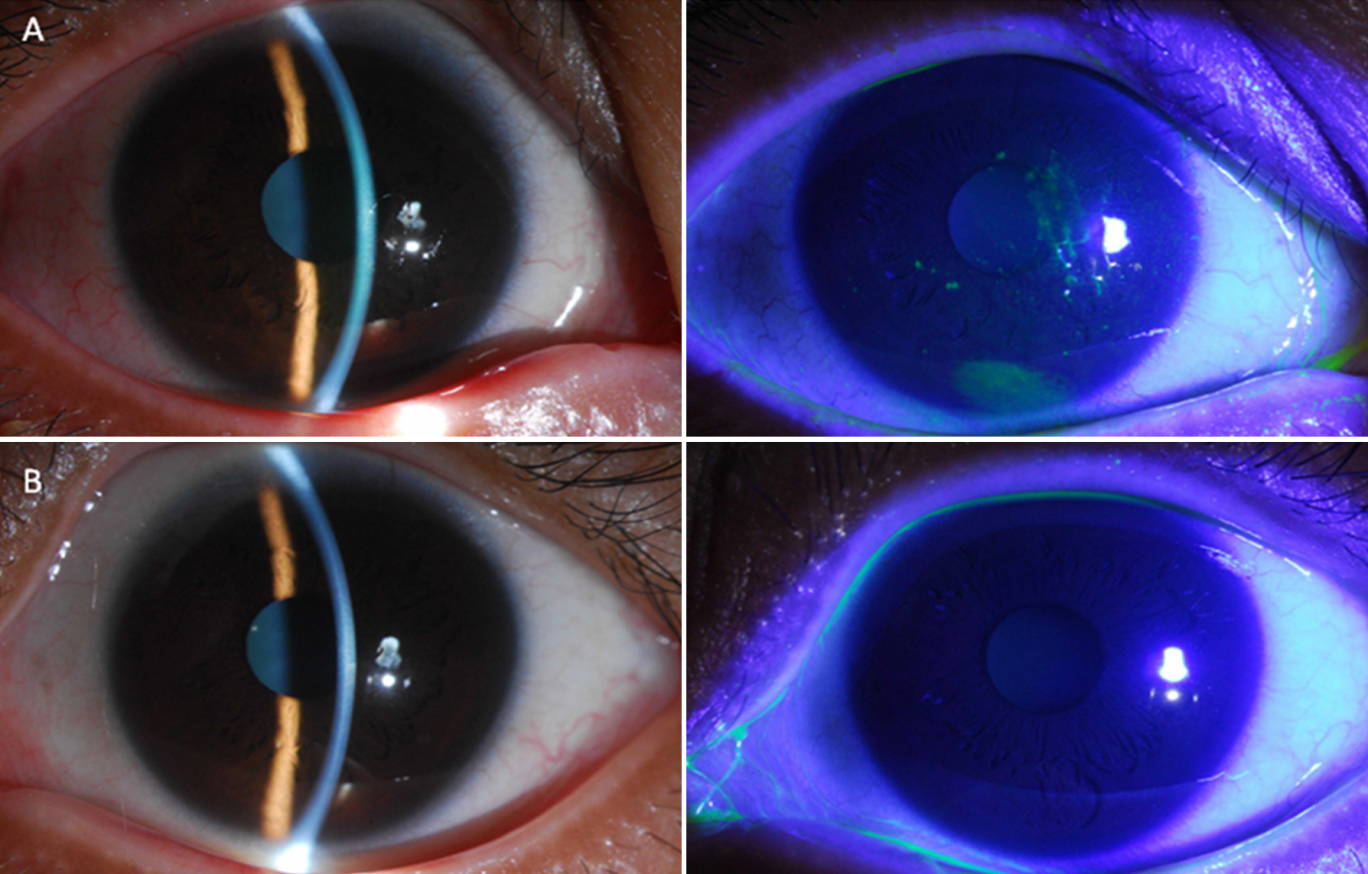
Corneal fluorescein staining photographs. Slit-lamp microscopic and fluorescein staining photographs from a representative patient with dry eye related to GVHD (**A**) and a control (**B**). Green punctate positive ﬂuorescein staining in **A** highlights the erosion of the corneal epithelium; **B** shows an intact corneal epithelium in a control.

Baseline subject characteristics are depicted in Table 2. There were no significant differences in age, sex, and primary disease before HSCT between both groups. As expected, levels of dry eye scores, OSDI scores, and Schirmer’s test scores were significantly increased in patients with dry eye related to cGVHD compared with the controls. As shown in Figure 1, fluorescein staining in a representative patient with dry eye related to cGVHD revealed obvious damage to the corneal epithelium compared with control patient.

**Table 2:** Baseline subject characteristics.

**Patient characteristics**	**Dry eye with cGVHD**	**Controls**	**p-values**
Male to female ratio	13:7	13:7	
Age	28.35±10.35	28.85±8.86	0.581
Primary disease before HSCT	ALL:3/AML:10 CML:7	ALL:3/AML:4 CML:11/MDS:2	
**Donor**			
Sibling	15	17	
Matched unrelated	5	3	
**cGVHD grade**			
I - II	9	NA	
III- IV	11	NA	
**Affected organ**			
Skin	16	NA	
Liver	8	NA	
Dry eye score	1.65±0.75	0	<0.001
OSDI score	31.85±8.11	73.25±18.78	0.047
Schermir test	3.72±1.54	7.55±2.68	0.001

Abbreviations: ALL=acute Lymphoblastic Leukemia; AML=acute myeloid leukemia; CML=chronic myelogenous leukemia; MDS=myelodysplastic syndrome; OSDI=Ocular Surface Disease Index; NA=not applicable.

### Expression of *TLR2* and its downstream molecules

**Figure 2:**
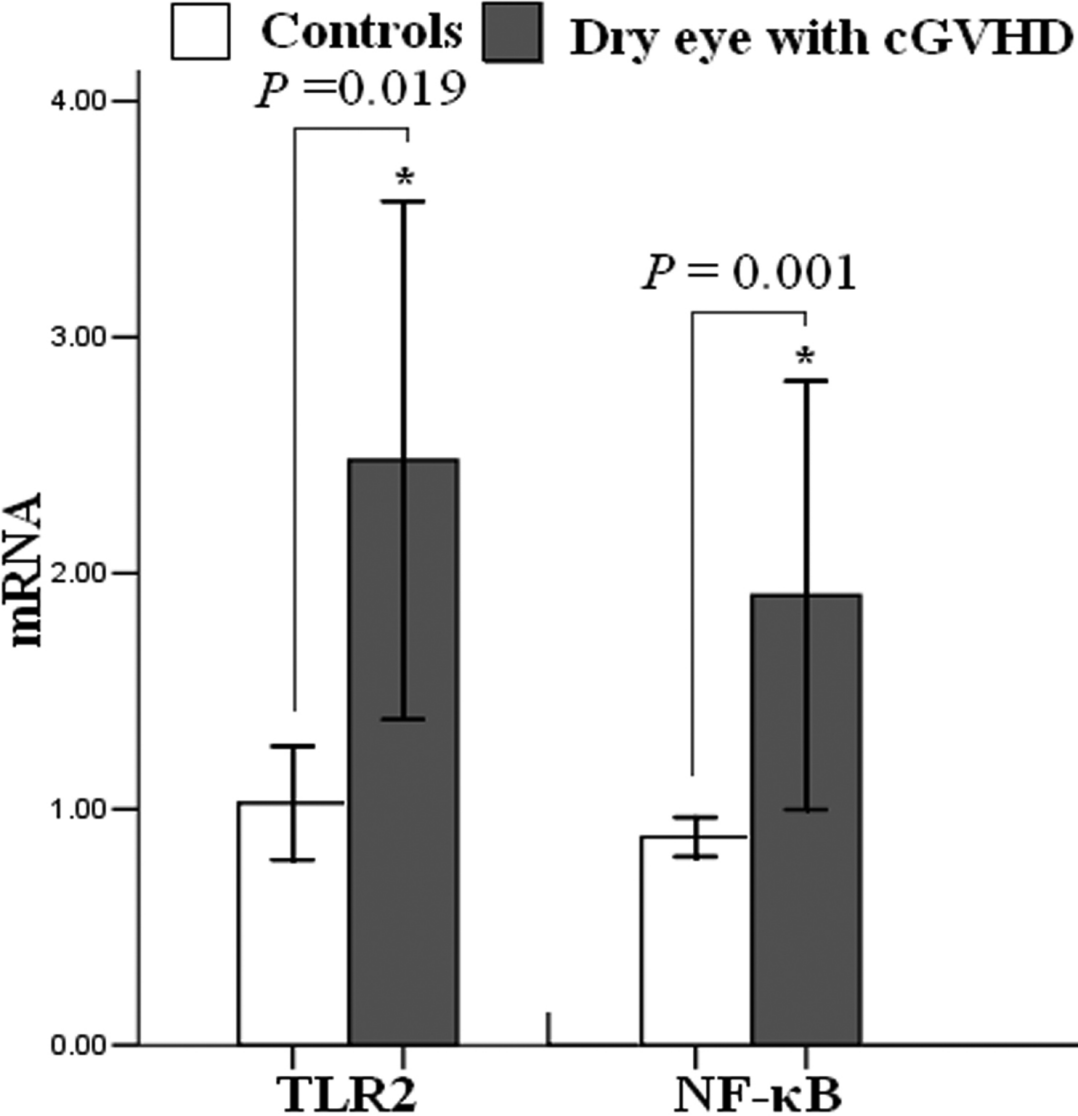
Expression of TLR2 and NF-κB in dry eye associated with cGVHD. Enhanced expression of *TLR2* and *NF-κB* was detected by qRT–PCR in dry eye related to cGVHD compared with the controls. Data from qRT–PCR are representative of three experiments and are presented as mean±SEM (p≤0.05).

Real-time PCR results revealed that *TLR2* expression showed a marked increase in patients with dry eye related to cGVHD (2.48±2.35 versus 1.03±0.51, p=0.019; Figure 2). The PCR analysis (1.91±1.94 versus 0.88±0.18, p=0.001) showed that *NF-κB*, the downstream gene of the TLR2 signaling pathway, was augmented in dry eye related to cGVHD (Figure 2).

### Production of TNF-α cytokine

**Figure 3:**
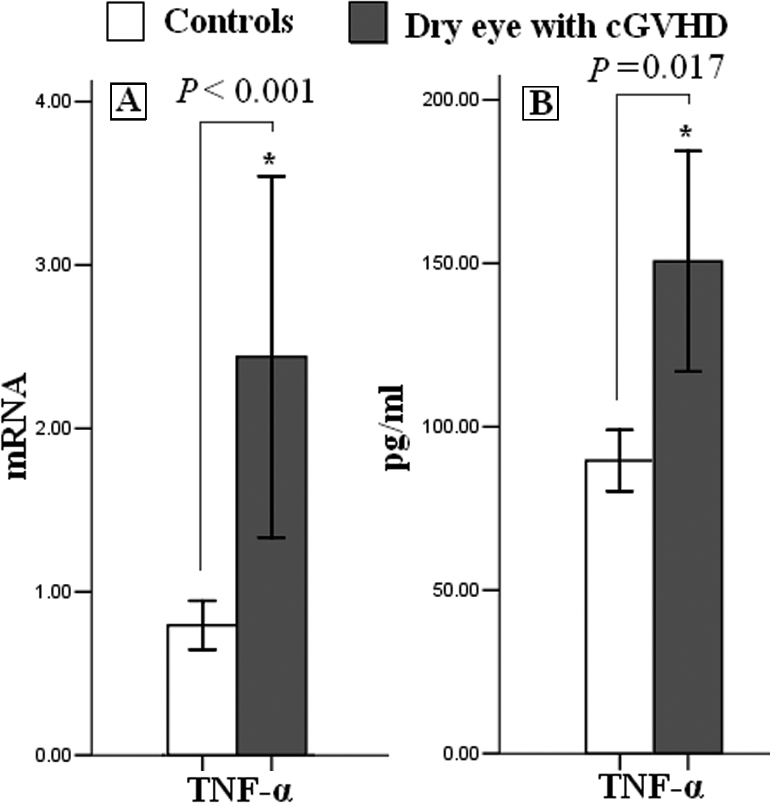
Increased *TNF-α* expression in patients with dry eye related to cGVHD. *TNF-α* expression was elevated both in the mRNA (**A**) and protein level (**B**) in dry eye related to cGVHD compared with the controls. Data are presented as mean±SEM (p≤0.05).

Stimulation of *NF-κB* can lead to enhanced expression of inflammatory mediators such as TNF-α. Therefore, we examined levels of *TNF-α* mRNA and protein secretion in patients with dry eye related to cGVHD and controls. As shown in Figure 3A,B, *TNF-α* mRNA levels were elevated in the patients with dry eye related to cGVHD (3.05±2.95 versus 0.99±0.40, p<0.001). The protein level of TNF-α detected by ELISA showed the same trend (225.86±108.14 pg/ml versus 134.44±29.294 pg/ml, p=0.017).

### Correlation analysis

There were significant positive correlations of *TLR2* expression with the OSDI scores and Schirmer’s test scores (r=0.565, p=0.010 <0.05; r=0.564, p=0.016 <0.05). In addition, NF-κB and TNF-α levels, the inflammatory pathway molecules, were also significantly correlated respectively with *TLR2* expression (r=0.509, p=0.022 <0.05; r=0.565, p=0.009 <0.05).

## Discussion

An inflammatory and fibrotic process in patients with cGVHD often produces debilitating dry eye syndrome [5-7]. cGVHD may also affect the skin and be associated with liver damage. There is increasing evidence that dry eye is accompanied by ocular inflammation characterized by increased expression of immune activation markers and infiltration of inflammatory cells in cGVHD patients that exhibit an abnormal immune reaction and systemic inflammation [5,8,9,23]. TLR2 signaling, mostly activated on APCs, has been considered essential for the inflammatory response and for immune disorders. In this study, we measured and analyzed alterations in the major components of TLR2 signaling in patients with dry eye related to cGVHD. Of these patients, 16 had cutaneous cGVHD with sclerodermoid features or erythema; eight had liver involvement with elevated liver function tests not explained by medication or other illnesses. However, we found that *TLR2* and its downstream targeted molecules were remarkably upregulated in patients with dry eye related to cGVHD. Especially, *TLR2* expression was significantly correlated with the dry eye profile such as OSDI scores and Schirmer’s test in these patients.

Several studies have investigated the markedly impaired immune function in the ocular surface of patients with dry eye related to cGVHD [8,24]. In fact, dry eye related to cGVHD is a systemic immune-inflammatory disorder. The local dry eye symptoms are secondary to the systemic disorders. Therefore, we focused on the systemic inflammatory response. To explore the molecular mechanisms underlying the induction of dry eye symptoms, we analyzed the expression of *TLR2* and the related downstream cytokines in peripheral blood. The findings relating to the activation of the TLR2 signaling pathway in systemic inflammation will provide new insight into the inflammatory process of dry eye associated with cGVHD. The up- or downregulation of these TLR2–NF-κB signaling molecules might serve as potential systemic indicators of the onset and progress of this disease when compared with local inflammatory markers detected by invasively scraping the conjunctival and corneal epithelium.

In recent years, TLRs have been reported to play a crucial role in amplifying autoimmune responses, ultimately resulting in inflammation [25]. Once TLR abnormalities occur, they may cause excessive inﬂammation due to either an immoderate innate immune response or to persistent compensatory adaptive immune activation [11]. *TLR2* activation has been reported to have the potential to exacerbate immune-inﬂammatory diseases in clinical and experimental settings. For example, the expression of *TLR2* and its ligands is elevated in macrophages from the joints of patients with rheumatoid arthritis [26]. Moreover, some novel findings suggest that TLRs may play major roles in the survival and maintenance of grafted organs [27,28]. When *TLR9* knockout mice were used as graft recipients, survival and pathological changes of acute intestinal GVHD were improved compared with the wild-type recipient mice [29]. Some researchers have reported that mutations in the *TLR4* gene were associated with an increased risk of severe acute GVHD [30,31]. However, the Goldstein group has suggested that *TLR2*^−/−^ but not *TLR4*^−/−^ recipient mice manifest a significant delay in graft rejection in a skin allograft model [32,33]. Furthermore, investigators have reported that *TLR2* stimulation can induce Th2 immunity [34,35], which is the prominent immunity of chronic GVHD, whereas *TLR9* stimulation promotes Th1 immune responses, the characterized immunity of acute GVHD [35]. Therefore, in this study we focused on *TLR2* to research the role of innate immunity in dry eye related to chronic GVHD. Our results pointed to the upregulated expression of *TLR2* and *NF-κB*, which efficiently trigger a cytokine storm and lead to the inflammatory response in patients with dry eye related to cGVHD.

*NF-κB* is a well known, classic inducible transcription factor of many signaling pathway, and it has been found to mediate the inflammatory role of TLR2 in several diseases [36]. Many studies have reported that TLR2 can activate *NF-κB*, termed the canonical pathway, to encode the inflammatory gene-like *TNF-α* [37]. Some preliminary trials have tried to regulate the inflammation of dry eye by neutralizing TNF-α [38,39]. However, the upstream molecules of this inflammatory signaling pathway have not been clarified. Exploring molecular mechanisms of TNF-α generation via the TLR2–NF-κB signaling pathway should be quite useful for developing therapeutic approaches to combat dry eye associated with cGVHD. As we expected, our results showed that there was a significant increase in the expression of NF-κB and TNF-α and that this was positively correlated with TLR2 upregulation. Thus, it is reasonable to conclude that TLR2–NF-κB signaling is activated in dry eye associated with cGVHD.

This preliminary study highlighted the potential role of TLR2 signaling in dry eye associated with cGVHD. Various mechanisms, such as how the TLR2–NF-κB signaling pathway is activated, the molecular events underlying APCs-T-cells interactions through TLR2 activation, and the inflammatory consequences of the activation of TLR2 on APCs need further extensive investigation. We are currently engaged in establishing a cGVHD model in TLR2 knockout mice to elucidate the molecular mechanism underlying the induction of cytokines/chemokines by TLR2 in dry eye associated with cGVHD.

In conclusion, this is the first report to suggest that *TLR2* and *NF-κB* expression is upregulated in dry eye associated with cGVHD and that this upregulation results in the significant generation of proinflammatory factor TNF-α. The TLR2–NF-κB signaling pathway contributes to the inflammatory state, which may predict the onset and progression of dry eye associated with cGVHD. Our findings provide new insights into the molecular mechanisms underlying the control of the innate immune response in the immunopathological processes of dry eye associated with cGVHD. These, in turn, should contribute substantially to a better understanding of the relationship between innate immunity and dry eye associated with cGVHD.
